# Posturography with head movements in the assessment of balance in chronic unilateral vestibular lesions

**DOI:** 10.1038/s41598-021-85745-x

**Published:** 2021-03-18

**Authors:** Magdalena Janc, Mariola Sliwinska-Kowalska, Piotr Politanski, Marek Kaminski, Magdalena Jozefowicz-Korczynska, Ewa Zamyslowska-Szmytke

**Affiliations:** 1 Audiology and Phoniatrics Clinic, Nofer Institute of Occupational Medicine, 8 St Therese Str., 91-348 Lodz, Poland; 2 Department of Radiological Protection, Nofer Institute of Occupational Medicine, Lodz, Poland; 3 Department of Microelectronics and Computer Science, Lodz University of Technology, Lodz, Poland; 4 Balance Disorders Unit, Department of Otolaryngology, Medical University of Lodz, The Norbert Barlicki Memorial Teaching Hospital, Lodz, Poland

**Keywords:** Diseases, Medical research

## Abstract

The aim of our study was to validate the method of head-shake static posturography (HS-posturography) in healthy individuals and to establish the value of this novel method in the diagnostics of patients with unilateral vestibular lesion (UV). The study included 202 participants divided into two groups, one consisting of 133 patients with canal paresis CP > 19% and one of 69 healthy subjects. Participant was tested according to the standard protocol of static posturography (SP), and with head movements of 0.3 Hz (HS 40), 0.6 Hz (HS 70) in random order controlled by a metronome. HS-posturography revealed a similar repeatability and internal consistency as the standard posturography. In patients with UV, 4th condition revealed higher sensitivity (74%) and specificity (71%) in HS 40 than in the standard posturography (67%, 65% respectively) and HS 70 (54%, 70% respectively). Static posturography and HS- posturography revealed a high reliability of the testing method. The head movements added to static posturography improve the sensitivity and specificity of the method in group with vestibular impairment. The most important test for that purpose seems to be the one on unstable surface with the eyes closed, with low frequency of head movements.

## Introduction

Static posturography is an essential test for assessing the balance system, predominantly the ability to keep the body in a steady upright position. Testing is performed both on a fixed and foam surface, with eyes open and closed. In quiet stance, the body is normally maintained upright and the center of mass projecting on the base of support provided by feet.

Since vestibular organ as a part of the balance system plays a minor role in the static balance, there is an effort to sensitize the classical static posturography method for vestibular input. For that reason, a test with the head movements added to this procedure is proposed.

To our knowledge, only one paper on static posturography with head movements was published so far by Cohen et al. in 2014^1^. The methodology used in this study significantly differed from the measurement used in the standard static posturography. Instead of a force plate, two inertial sensors were attached on the patient’s back at mid-thoracic level and on their head. The tests were performed on the floor and on a foam, with the head kept still or with it moving with the frequency of about 0.33 Hz in the pitch and jaw (horizontal) planes. The ability to keep balance during 30 s. the number of head movements during that time were the main outcomes of the study. Although the body sways measured by the inertial sensors were significantly higher in the group with balance problems as compared to the control group when the head was moving in the pitch plane, for a subgroup of patients with unilateral vestibular weakness the worst results were observed for jaw plane movements.

There is a sparse literature data concerning head shaking [HS] in relation to the Sensory Organization Test (SOT) of the computerized dynamic posturography. The pilot study by Shepard and Speers revealed an increase in the number of sways after adding the head movements to classical testing in patients with peripheral vestibular impairment, who initially presented normal SOT results^2^. Since then, head-shake dynamic posturography has been recognized as an indicator of incomplete dynamic compensation in subjects with canal paresis, in whom a static compensation was reached^3^. After promising preliminary results, a number of studies have been performed to determine the optimal test parameters. Two out of the six standard SOT conditions, SOT 2 and SOT 5 have been chosen for head-shake posturography (HS-posturography)^4–8^. In most cases, the horizontal plane was used to move the head, which means the same kind of movement as in the rotatory chair tests and in the caloric assessment^4,5^. The range of motion should be safe for patients with cervical spine degeneration; thus, Mishra and Honaker suggested an angle of 15° to the right and to the left from the central position, which is available for subjects with reduced mobility of the cervical spine^4,5,9^. Velocity and frequency of head movements seem to have the greatest influence on the test results. At the beginning, Mishra et al. used approximately 1 Hz frequency but a high percentage of falls was found in the group of people with vestibular disorders. Honaker et al. introduced head movements with peak velocities of 15°/s, 60°/s and 120°/s. In a group of healthy volunteers, the results revealed differences between HS and standard posturography for each velocity depending on the test^5^. Finally, the velocity of 15°/s was classified as the most useful for patients with vestibular impairment^9^. The sensitivity and specificity of HS-SOT 5 has been calculated as 70% and 100% respectively.

Although the value of HS computerized dynamic posturography is quite well recognized in the diagnostics of patients suffering from vestibular lesion, the examination requires advanced equipment usually not available in a general practitioner’s office. The HS added to static posturography, which requires a much less expensive platform, may be the preferable choice in such cases.

The aim of this study was to validate the method of head-shake static posturography (HS-posturography) in healthy individuals and to establish the value of this novel method in the diagnostics of balance abnormalities in patients with chronic unilateral vestibular lesion.

## Materials and methods

### Control group

The healthy reference group (H group) included 69 healthy students and office workers, 30 women and 39 men, aged from 20 to 74 years (mean age 43.1; SD 17.1 years) recruited voluntarily by advertisement.

The inclusion criteria for H group were the following:

- in anamnesis: no vertigo, dizziness, unbalance, any known neurological, circulatory and musculoskeletal system disorders, diabetes, and migraine;
- no signs of central or peripheral vestibular abnormalities in physical examination and videonystagmography (VNG) testing;

The normal score of the Dizziness Handicap Inventory (DHI) was noticed in the group (mean results 4.5 points).

A subgroup of six healthy people was formed to assess the repeatability and reliability of HS posturography results. This subgroup consisted of one man and five women, aged 43.5 (SD 8.8 years).

### Study group

The study group included 133 patients, 92 women and 41 men, diagnosed in the Audiology Clinic with unilateral peripheral vestibular weakness (UV group). The age ranged from 21 to 79 years (mean age 53.7; SD 13.75 years).

All subjects underwent audiological (otoscopy, hearing tests) and neuro-otological examinations including history of disease, physical examination (eye-movement examination: spontaneous, gaze evoked nystagmus, convergence, pursuit, saccades, head impulse test; Romberg and Unterberger tests) and videonystagmography (VNG) recordings. Caloric testing: bithermal (44 °C and 30 °C) water caloric test was performed, where Ulmer videonystagmography was used to record the response. Caloric reactivity (the summed caloric responses in the best responding ear) and asymmetry were calculated by the device. CP was considered normal if the difference between both ears was less than 20%, based on a normative study performed in the same clinical setting, with exactly the same VNG procedure. Sinusoidal Harmonic Acceleration (SHA) tests were performed using the Ulmer motorized chair, with testing frequencies 0.04, 0.08, 0.1, 0.32, 0.64 Hz.

The inclusion criteria for this group were the following:

- asymmetry in the function of vestibular organs, as assessed by the videonystagmography (VNG) caloric test (canal paresis CP > 19%, reactivity > 20°/s);
- no signs of central abnormalities of the balance system: normal saccades, smooth pursuit and kinetic test results in VNG; nystagmus, if observed, corresponding to Alexander’s law in clinical examination;
- no signs of benign paroxysmal positional vertigo in anamnesis during last six months and a negative result of the Dix-Hallpike positional test in clinical examination;
- absence of neurological, circulatory and musculoskeletal system disorders, no diabetes, no migraine;

The study group patients were diagnosed with vestibular neuritis; there were no subjects with acute vestibular vertigo in the group. The outpatient clinic is intended for patients in subacute or chronic status, at the earliest two months after the vestibular onset.

Only about 25% of patients revealed mild to moderate disability in Dizziness Handicap Inventory (mean score 42.64 points, SD 22.2), in VNG caloric 24% of subjects recovered and reveled CP in range 20–24%, the remaining patients’ caloric asymmetry was ≥ 25%.

The research protocol was approved by the Bioethics Commission affiliated to the Nofer Institute of Occupational Medicine, Lodz, Poland (No. of protocol 12/2017). Each subject gave written consent to participate in the study and had their data processed in accordance with the Helsinki Regulation and European Union directives on the protection of personal data of 25 May 2018.

## Methods

The NeuroCom Static Balance Master posturography equipment and the modified Clinical Test of Sensory Interaction on Balance (mCTSIB) protocol were used in all subjects of this study. According to the mCTSIB, subjects were asked to stand with their hands at their sides, feet apart, and fulfill the following 4 sensory conditions:

1st—stand on firm surface, eyes open;

2nd—stand on firm surface, eyes closed;

3rd—stand on foam surface, eyes open;

4th—stand on foam surface, eyes closed.

Each condition consisted of three ten-second trials, the mean values of three trials were calculated. The mCTSIB protocol was performed three times. The first one was standard (SP), then the protocol was repeated with the head moving with the frequency of 0.3 Hz (HS 40 meaning 40 beats per minute by metronome), in the range of 15° to the right and to the left from the central position and then with the frequency of 0.6 Hz (HS 70 meaning 70 beats per minute) in the same range.

Before testing, subjects were taught the rhythm of head movements. To facilitate the task, three dots were marked on the wall in front of the examined person: one in central position, one at 15˚ to the right and one 15˚ to the left from the center. The subject was asked to move their head in the rhythm of the metronome and fix their eyes on the dots. Then the dots were removed and the tests started. Thus, the test was performed without eye fixation, which means that neither in open-eyes conditions nor in close-eyes ones patient was not obliged to keep fixation at any point. The first protocol was always the standard mCTSIB without head movements, then the protocol was repeated twice with the head movements. HS 40 and HS 70 tests were performed in random order. The head movements were executed in accordance with the metronome beats. The foam which was used in the 3rd and 4th conditions was dedicated to the NeuroCom device. The foams dimensions were the following: 45 × 45 × 12.3 cm with density of 64 kg/m^3^.

The average value of angular velocity was calculated by the device software with an accuracy of 0.1 [°/s]. Moreover, the compositum (Comp) coefficient was calculated as the average value of all four conditions. When the subject was not able to keep balance during the test, their fall was automatically marked by the software and the value of 6˚/s was used for calculation.

For repeatability of the assessment, all testing protocols were repeated eight times during consecutive days. The time of the day and tests order differ from day to day.

### Statistics

Repeatability and accuracy of the HS posturography were assessed and compared to the standard static posturography. The level of accuracy of measurement method was assessed in agreement with ISO 5725-2 procedure "The basic method of determining repeatability and reproducibility of the standard measurement method”^10^. Standard deviation was the measure of precision. Repeatability was calculated as a percentage of the coefficient of variation (cvsr) according to the formula:

$$ {\text{SD}}_{{\text{r}}} = \sqrt {\frac{1}{n - 1}\mathop \sum \limits_{i = 1}^{n} \left( {x_{i} - x} \right)^{2} } ,\quad {\text{csvr}} = \frac{{SD_{r} }}{x} \cdot 100\% $$

n: number of measurements, x: mean value, x_i_: i measured value, SDr: standard deviation repeatability, csvr: coefficient repeatability.

The reliability was calculated based on the intraclass correlation coefficient (ICC) and Cronbach’s alpha for tests repeated eight times for six healthy participants separately in every method. The ICC and Cronbach’s alpha value > 0.9 were assumed to be excellent^11^.

$$ {\text{ICC}} = \frac{{MS_{R} - MS_{E} }}{{MS_{R} + \frac{{Ms_{c} - MS_{E} }}{n}}} $$

n: number of subjects; MS_R_: mean square for rows (six subjects); MS_E_: mean square error, MS_C_: mean square for columns (three trials during 8 days).

Normative values for HS posturography were established based on the data received from the 69 healthy volunteers. The correlation with the age was estimated using the Spearman’s correlation coefficient, the meaning of which was interpreted according to Gill-Body et al.^12^. The values < 0.25 were considered to be weak, values 0.26–0.50—fair, values 0.51–0.75—moderate and values of ≥ 0.76 were considered to indicate a strong relationship. For further analyses, the healthy group was divided into three subgroups aged 20–39, 40–59 and above 60 years. The results were compared using one-way analysis of variance (ANOVA) and the Levene's variance. To test the differences between the groups, multiple comparisons were performed. Scheffe or Tamhane post-hoc tests were used depending on the Levene’s test result. Age-dependent values were calculated according to the model—mean value + 1 standard deviation.

To compare the posturography results between the UV group and the healthy one, the multivariate ANCOVA analysis was used. Age was the quantitative variable. The ROC curves were used to establish the sensitivity and specificity of posturography methods in unilateral weakness group. To avoid the ceiling effect, the number of maximum values (falls) was calculated and should not exceed 15%^13^.

The criterion of statistical significance p < 0.05 was used in statistical analyses. IBM SPSS Statistics 22 was used.

### Ethical approval

All procedures performed in studies involving human participants were in accordance with the ethical standards of the institutional and/or national research committee and with the 1964 Helsinki Declaration and its later amendments or comparable ethical standards. The study was approved by the Bioethics Committee of the Nofer Institute of Occupational Medicine, Lodz, Poland (No of protocol 12/2017).

## Results

### Repeatability and accuracy of Head Shaking posturography (HS posturography)

SP was the reference method. The values of HS 40 and HS 70 were subtracted from SP and the result of 5% was not exceeded for any condition (Table 1). The percentage coefficient of variation was in the range 11–20% (Table 1). Intraclass correlation coefficients were in ranges 0.70–0.96 (Table 2). In SP and HS 70 ICC coefficients were lower for 1^st^ condition.

**Table 1:** Coefficients of variability of repeatability for individual tests and methods.

Method	Conditions	SDr	cvsr (%)	Method tested-reference method [%]
SP	1	0.04	20	Reference
	2	0.05	18	
	3	0.06	14	
	4	0.13	14	
	Comp	0.05	11	
HS 40	1	0.04	18	− 2
	2	0.05	17	− 1
	3	0.05	11	− 3
	4	0.16	17	3
	Comp	0.06	11	0
HS 70	1	0.05	20	0
	2	0.05	16	2
	3	0.08	14	0
	4	0.16	15	− 1
	Comp	0.05	10	1

*SP* static posturography, *HS 40* posturography with head frequency movements 0.33 Hz, *HS 70* posturography with head movements at a frequency of 0.58 Hz, *SDr* standard deviation of repeatability, *cvsr* coefficients of variability determining repeatability.

Conditions: 1 (eyes open/stable surface), 2 (eyes closed/stable surface), 3 (eyes open/unstable surface), 4 (eyes closed/unstable surface) and comp (average value of all 4 tests as a coefficient equilibrium).

**Table 2:** Intraclass correlation coefficient (ICC) in study methods.

	Condition 1	Condition 2	Condition 3	Condition 4
SP	0.7	0.96	0.91	0.91
HS 40	0.92	0.93	0.94	0.93
HS 70	0.79	0.93	0.95	0.95

Conditions: 1 (eyes open/stable surface), 2 (eyes closed/stable surface), 3 (eyes open/unstable surface), 4 (eyes closed/unstable surface).

*SP* static posturography, *HS 40* posturography with head frequency movements 0.33 Hz, *HS 70* posturography with head movements at a frequency of 0.58 Hz.

### Normative values of HS posturography by age

The moderate or weak Pearson’s correlations between age and posturography results were observed in the group of healthy people (n = 69). The correlation coefficients did not reach the statistical significance for the 4th condition in SP and all conditions in HS 70 (Table 3). However, when the volunteers were divided into three age groups: 20–39 years old, 40–59 years old and over 60 years old, ANOVA and post-hoc tests revealed significant differences between utmost groups in standard posturography and HS 40 posturography. Table 3 presents the normative values (n = 69) of sway angular velocities calculated as a mean value plus standard deviation, which are proposed for age ranges.

**Table 3:** Normative values of tests for each method in age groups.

Age	SP					HS 40					HS 70				
	1	2	3	4	comp	1	2	3	4	comp	1	2	3	4	comp
20–39 n = 40	0.2^a^	0.4^a^	0.6^a^	1.8	0.7^a^	0.3^a^	0.5	0.8^a^	1.8^a^	0.8^a^	0.4	0.5	1.2^c^	2.4	1.1
40–59 n = 11	0.3	0.4	0.7^b^	2.0	0.8	0.3	0.5	0.8	2.0	0.9	0.4	0.5	1.1	2.3	1.0
60 ↑ n = 18	0.3	0.5	0.8	2.1	0.9	0.4	0.7	1.0	2.3	1.1	0.4	0.6	1.2	2.5	1.1
Cor. coeff	0.5	0.39	0.57	0.18	0.44	0.35	0.32	0.34	0.36	0.42	0.19	0.23	0.16	0.06	0.03

Conditions: 1 (eyes open/stable surface), 2 (eyes closed/stable surface), 3 (eyes open/unstable surface), 4 (eyes closed/unstable surface) and comp (averaged value from all 4 tests as a coefficient equilibrium).

*SP* static posturography, *HS 40* posturography with head movements at a frequency of 0.33 Hz, *HS 70* posturography with head movements at a frequency of 0.58 Hz, *Cor. Coeff.* correlation coefficient between age and posturography results.

The statistically significant differences between groups: ^a^group20-39 vs group 60 + , ^b^ group 40-59vs group 60 + , ^c^group 20-39vs group 40–59; (p < 0.05).

### Sensitivity and specificity of HS posturography for unilateral vestibular impairment

In the unilateral weakness (UV) group, the percentage of maximum sway value did not exceed 15% of the results in every method. Thus, no ceiling effect was observed.

**Figure 1:**
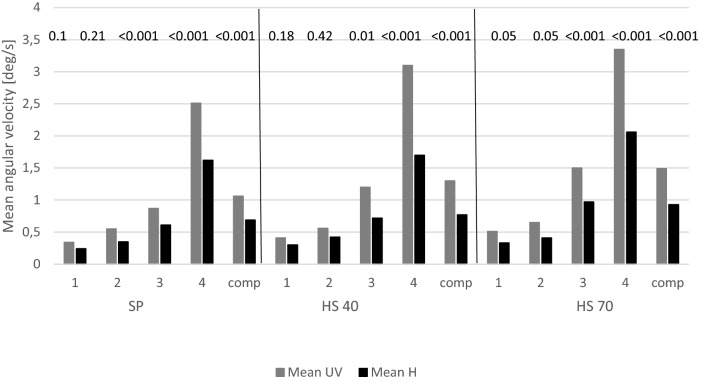
Weighted average values of sway's angular velocities in the UV group and healthy group. *SP* static posturography, *HS 40* posturography with head movements at a frequency of 0.3 Hz, *HS 70* posturography with head movements at a frequency of 0.6 Hz; Conditionss: 1 (eyes open/stable surface), 2 (eyes closed/stable surface), 3 (eyes open/unstable surface), 4 (eyes closed/unstable surface) and Comp (averaged value from all 4 tests as an equilibrium coefficient), mean UV—weighted average sway's angular velocities in the group with unilateral vestibular weakness; mean H—weighted average sway's angular velocities in the group of healthy people.

The mean angular sway velocities were compared between the group of 133 people with unilateral vestibular lesions (UV) and the group of 69 healthy people Age was significantly different in two groups (Mann–Whitney U test, p < 0.001), multivariate ANCOVA analysis was used. The statistically significant differences between the groups were noticed for the third and 4th condition and the composite coefficient in every method (Fig. 1).

Finally, the sensitivity and specificity of individual condition of every test was determined using ROC curves, which along with the cut-off points are shown in Table 4. Condition 4 revealed highest sensitivity (74%) and specificity (71%) in the HS 40 than in the standard posturography (67%, 65% respectively) and the HS 70 (54%, 70%, respectively).

**Table 4:** Summary of ROC curves analysis for individual tests of each methods in differentiating patients with UV from healthy group.

Method	Conditions	Sensitivity	Specificity	AUC	Cut-point [deg/s]
SP	1	74	57	**0.70**	0.2
	2	77	39	0.68	0.3
	3	74	71	**0.78**	0.6
	4	67	65	**0.75**	1.7
	Comp	68	83	0.80	0.8
HS 40	1	73	40	0.63	0.3
	2	71	35	0.59	0.3
	3	75	71	**0.79**	0.8
	4	**74**	**71**	**0.80**	1.8
	Comp	74	83	0.80	0.8
HS 70	1	73	44	0.66	0.3
	2	73	49	0.68	0.4
	3	60	70	0.68	1.0
	4	54	70	0.67	2.2
	Comp	55	81	0.71	1.1

Conditions: 1 (eyes open/stable surface), 2 (eyes closed/stable surface), 3 (eyes open/unstable surface), 4 (eyes closed/unstable surface) and comp (averaged value from all 4 tests as the balance factor).

*SP* static posturography, *HS 40* posturography method with head movements at a frequency of 0.33 Hz, *HS 70* posturography method with head movements at a frequency of 0.58 Hz, *AUC* value of the area under the ROC curve.

p < 0.05.

## Discussion

Head shake posturography is a novel test for which no methodology has been established so far. The main objective of the study is to introduce the method of balance assessment during vestibular stimulation contrary to the static and dynamic posturography in which the head is kept still. The previous studies used the dynamic posturography protocol as a base for the head shaking posturography. Although the static posturography partly resembles the dynamic one, static posturography in some ways is more challenging for patients, which increases its clinical utility^1^. Furthermore, static posturography is portable and less expensive than dynamic posturography, which makes it more suitable for screening. For that reason, static posturography was used in the study.

### Methodology, repeatability and accuracy of Head Shaking posturography (HS posturography)

The main issue associated with introducing the new method is choosing the parameters of head movement that would be the most suitable for revealing the influence of vestibular impairment on the balance system. In our study, two frequencies of head movements, 0.3 Hz (HS 40) and faster 0.6 Hz (HS 70) were compared. The testing frequencies belong to the medium range of vestibular stimulation, higher than the caloric test, but lower than head shaking. The main concern being that too rapid movement may cause subjects to fall and too slow movements may not activate the vestibular system, particularly in compensated subjects. In literature data, the first choice was the head movements within the frequency of 1 Hz^4^. These vigorous head movements appeared to be too difficult for vestibular patients who frequently failed in SOT 5. Honaker et al. proposed three head peak velocities: 120°/s, 60°/s and 15°/s which can be calculated, taking into consideration the movement range of 15° to the right and left, as 1.28 Hz, 0.64 Hz and 0.16 Hz, respectively^5^. The head movements of 0.16 Hz and 0.64 Hz were applied to SOT 5 and 0.64 Hz and 1.28 Hz were used in SOT 2. In vestibular subjects, 0.16 Hz in SOT 5 test was found as the most appropriate. However, SOT 5 (eyes closed, the moving platform) is not easily reproduced in static posturography, where the test on the foam seems to be more challenging for patients. Moreover, three frequencies of head movements require a longer training which significantly impedes the examination. For that reason, only two frequencies were used in our study, and fast movements which were less reliable and more uncomfortable for the patient were abandoned.

In the first part of the study, the reliability of the test method was assessed^14^. Based on the ISO standard, the repeatability of static posturography (SP) and HS posturography methods were evaluated. The ISO standard assumes that the difference in repeatability coefficient between the method tested and the reference one should not exceed 5%, and this condition was fulfilled. The variability that we calculated was similar to the range of 9–15% which was suggested by Di Berardino et al.^15^.

In general, the internal consistency of tests repeated in consecutive days was high for all methods. However, there were slightly worse results in the first condition (firm, eyes open) in static posturography and HS 70. Lower reliability of the results in the first condition of static posturography was also shown by Hill et al.^16^. This may be explained by the low resolution of this method (0.1°/s), which is comparable to the results of the healthy group in this test (0.2°/s).

Adding the head movements to the standard static posturography did not influence the high repeatability of the test. It should be noted that in our study the highest intraclass correlation coefficients were observed for HS 40 and only for this method the ICC criteria (> 0.9) were met for all conditions.

We have found only one study in which the reliability of the method with head movements has been assessed. Pang et al. used two tests from the protocol of dynamic posturography: SOT 2 and SOT 5 with head movements of approximately 1 Hz frequency. They tested 77 healthy volunteers divided into two age groups: below 50 years (mean 24.2) and over 50 years old (mean 58.0). They noticed lower inter-class correlation coefficients in the older group (0.64 and 0.55) as compared to the younger one (0.85 and 0.78), which suggests a lower reliability of the test results in an older population^7^. In our study the relationships between age and the results of posturography tests were moderate and weak for SP and HS 40, and very weak for HS 70. A statistical significance was found between the utmost age groups in SP and HS 40 only. The main disadvantage of the study was the age range of the healthy group. Although many older people were recruited, they reported systemic heart or musculoskeletal disorders which excluded their participation. In modified static posturography tests, Cohen et al. found a similar age dependence between the youngest and the oldest participants but Agrawal et al. observed more linear increase in sways with age in the range from 40 to over 80 years^17,18^. For HS posturography, Park et al. noticed linear relationships between age and HS SOT 5, while HS SOT 2 revealed the difference between the youngest and the oldest groups^6^. Pang et al. showed significant differences between group subjects < 50 and > 50 years old in HS SOT 5 with 1 Hz head movements^7^. Also, in the study of Honaker et al. there was a certain trend above the age of 70^9^. Taking into consideration our results and the literature data, the normative values proposed in our study were age-referenced.

### Sensitivity and specificity of HS posturography for unilateral vestibular impairment

The main aim of the study was an assessment of the utility of HS methods for diagnostics of balance abnormalities in patients with unilateral vestibular weakness. Study group included patients, who were diagnosed with vestibular neuritis and still suffer from vertigo and imbalance. Caloric test results were the main criterion to compose vestibular group. Since patients were in subacute or chronic stage of the disease, the cutoff point of 20% was used to recognize vestibular asymmetry, similarly as in Cohen’s study^1^. In the group with partial compensation a high sensitivity of the posturography was not expected. Standard static posturography is known with low sensitivity and higher specificity for the diagnosis of chronic unilateral peripheral vestibular lesions. In Di Fabio study sensitivity was calculated as 53% and specificity as 90%^19^, in El-Kashlan et al. study values were 60% and 87%, respectively^3^.

Initially, dynamic posturography has been proposed as a method more challenging for vestibular system by disrupting sensory reactions. However, the sensitivity appeared to be as low as 40% with specificity 90% in Di Fabio study^19^ or 73% and 40%, respectively, for SOT 5 in Mishra paper^4^. Thus, the attempts of adding the head movements were performed for dynamic posturography by Mishra et al.^4^ and Honaker et al.^9^. In the study of Mishra et al., the sensitivity of SOT 5 was improved to the value of 95% but the specificity of the HS test was very poor (22%). Honaker et al. achieved the higher sensitivity of 70% and excellent specificity of 100% for the slow head movements in SOT 5^9^, however, the small number of participants which were clinically heterogeneous requires careful interpretation of their results.

The only studies on head movements static posturography were performed by Cohen et al.^1,20^. They performed tests on a foam with eyes closed (4th condition of mCTSIB) and their main outcomes were the timing of and the ability to make at least 5 cycles of head movements. The kinematic data from the torso-mounted inertial motion sensor was also analyzed but differences between control and vestibular patients have not been found^20^.

In our study the mean sways velocity differed significantly between the vestibular and healthy groups in conditions on the foam both for standard posturography and HS posturography. Although the highest sensitivity and specificity for vestibular damage was observed in 3rd condition of HS40, these values were similar in standard and HS posturography. Adding slow head movements to standard posturography increases the sensitivity and specificity of the 4th condition (on foam with eyes closed) which condition is characteristic for vestibular impairment assessment. In contrary, 4th condition of the test with fast head movements revealed the lowest sensitivity. The possible explanation may include the multi-task nature of head movements posturography. Fast head movements are more challenging for vestibular system, which is important for uncompensated patients. However, slow movements require more concentration than rhythmic fast movements, which, along with the motor task, may be more difficult, particularly for older population^21^.

Age difference between study groups was the weak point of the study. As mentioned above, there was not enough number of older people without concomitant diseases recruited to the healthy group despite applying the same criteria to the healthy and vestibular groups. The heterogeneity of the patient’s compensation degree is the next weak point. Further research is needed to assess the influence of head movements on balance impairment in patients homogeneous in terms of compensation.

## Conclusions

Static posturography and head shaking posturography revealed high reliability of the testing method. The head movements added to static posturography improve the sensitivity and specificity of the method in group with vestibular impairment. The test on an unstable surface with eyes closed and added the low frequency head movements seems to be most important for that purpose.

## Data Availability

The datasets generated during and/or analyzed during the current study are available from the corresponding author on reasonable request.
